# A Novel Alpha Cardiac Actin (*ACTC1*) Mutation Mapping to a Domain in Close Contact with Myosin Heavy Chain Leads to a Variety of Congenital Heart Defects, Arrhythmia and Possibly Midline Defects

**DOI:** 10.1371/journal.pone.0127903

**Published:** 2015-06-10

**Authors:** Céline Augière, Simon Mégy, Rajae El Malti, Anne Boland, Loubna El Zein, Bernard Verrier, André Mégarbané, Jean-François Deleuze, Patrice Bouvagnet

**Affiliations:** 1 EA 4173, Université Lyon 1 and Hôpital Nord-Ouest, Lyon, France; 2 IBCP, UMR 5305 CNRS and Université Lyon 1, Lyon, France; 3 Laboratoire Cardiogénétique Malformation, Centre de Biologie et Pathologie Est, Hospices Civils de Lyon, Bron, France; 4 Centre National de Génotypage, Evry, France; 5 Unité de Génétique Médicale, Faculté de Médecine, Université Saint-Joseph, Beirut, Lebanon; 6 Institut Jérôme Lejeune, Paris, France; 7 Service de Cardiologie Pédiatrique, Hôpital Louis Pradel, Hospices Civils de Lyon, Bron, France; Loyola University Chicago, UNITED STATES

## Abstract

**Background:**

A Lebanese Maronite family presented with 13 relatives affected by various congenital heart defects (mainly atrial septal defects), conduction tissue anomalies and midline defects. No mutations were found in *GATA4* and *NKX2-5*.

**Methods and Results:**

A set of 399 poly(AC) markers was used to perform a linkage analysis which peaked at a 2.98 lod score on the long arm of chromosome 15. The haplotype analysis delineated a 7.7 meganucleotides genomic interval which included the alpha-cardiac actin gene (*ACTC1*) among 36 other protein coding genes. A heterozygous missense mutation was found (c.251T>C, p.(Met84Thr)) in the *ACTC1* gene which changed a methionine residue conserved up to yeast. This mutation was absent from 1000 genomes and exome variant server database but segregated perfectly in this family with the affection status. This mutation and 2 other *ACTC1* mutations (p.(Glu101Lys) and p.(Met125Val)) which result also in congenital heart defects are located in a region in close apposition to a myosin heavy chain head region by contrast to 3 other alpha-cardiac actin mutations (p.(Ala297Ser),p.(Asp313His) and p.(Arg314His)) which result in diverse cardiomyopathies and are located in a totally different interaction surface.

**Conclusions:**

Alpha-cardiac actin mutations lead to congenital heart defects, cardiomyopathies and eventually midline defects. The consequence of an *ACTC1* mutation may in part be dependent on the interaction surface between actin and myosin.

## Introduction

The prevalence of congenital heart defects (CHD) is about 0.8% of live birth and higher in still birth. The etiology of CHD is complex and combines both environmental and genetic causes. However, there are already more than 50 genes associated with CHD in humans [1]. Familial cases of CHD were very useful to discover most of these CHD genes. Although these discoveries concern a very small percentage of CHD cases, they shed a new light on cardiac diseases because they demonstrated in several cases that mutations in a single gene can result in CHD, arrhythmia and/or cardiomyopathies. Thus, mutations in *TBX20* [2,3], *MYH7* [4,5], *NKX2-5* [6,7], *GATA4*[8], and *ACTC1* [9,10] lead to a variety of cardiac anomalies including CHD, arrhythmia and cardiomyopathies. It is also the case in Noonan syndrome—now part of a larger group of disease referred to as RASopathies—where CHD and hypertrophic cardiomyopathies are found [11,12]. This is not yet the case of all cardiac genes but it showed that genes which are important for cardiac development might also be important for cardiac function during adulthood [6,13]. It is not clear yet why mutations in a single gene results in such diverse cardiac anomalies. It could either be related to modifier genes when a single mutation results in various cardiac diseases or it could be due to diverse protein dysfunctions when different mutations in a single gene result in various cardiac anomalies.

In this study, we report on a large Lebanese family with 13 affected members suffering from various congenital heart defects, arrhythmia, and valvular and conduction anomalies. In addition, several cardiac patients have also midline defects. A missense mutation was found in the alpha-cardiac actin gene which cosegregated perfectly within the family. This mutation (p.(Met84Thr)) and 2 other mutations also responsible for CHD (p.(Glu101Lys) and p.(Met125Val)) mapped to a small domain of the actin protein in very close contact to the myosin heavy chain, suggesting that disruption of this interaction domain leads to an altered cardiac development.

## Materials and Methods

### Patients

Members of this Lebanese family were examined by a cardiologist (clinical examination, rest ECG and echocardiography) and by a geneticist (clinical examination). After signing an informed consent, a peripheral blood sample was obtained to extract DNA with a standard protocol. The study was approved by the ethical committee of Hôtel Dieu hospital, Beirut, Lebanon.

### Genotyping and linkage analysis

Genotyping from all available DNA samples was performed at the Genotyping National Center (CNG, Evry). A panel of 399 microsatellites was tested (Life Technologies, Evry, France). Multipoint linkage analysis was prepared with easyLINKAGE [14] and performed with GeneHunter v2.1r5 [15] initially with a disease allele frequency of 0.01%, a fully penetrant disease and a 0% phenocopy rate and then with decreasing penetrance values to 85%. Haplotypes of the region of interest were prepared with GeneHunter. Genehunter output files were used to visualize haplotypes with the Haplopainter 1.043 software [16].

### DNA sequencing

The sequence of *NKX2-5* (ENST00000329198) and *GATA4* (ENST000335135) exons was obtained by Sanger sequencing. The 6 coding exons of the *ACTC1* gene (ENST00000290378) were amplified by PCR with 60ng of genomic DNA, 1.5 mM of MgCl2, 0.5 μM of forward and reverse primers, 0.2 mM of dNTPs and 1 U of Taq Platinum DNA polymerase (Invitrogen, San Diego, CA, USA) with appropriate buffer. PCR products were purified with the NucleoFast 96 PCR Clean up kit (Macherey-Nagel). Sanger sequencing was done with 0.8 μL BigDye terminator V1.1 or V3.1 with the appropriate buffer, 0.5μM of each primer and 1μL of PCR product denatured at 95° for 1 minute, then 25 cycles at 95° for 1 min, 50° for 1 min and 60° for 4 min 30 s. X-terminator product purification was done before it was sequenced with an Applied Biosystems 3730 DNA Analyzer. Sequence analysis was carried out with SeqScape v2.5 software. Variant analysis was carried out with Visual Alamut 2.6.1 Software. The variant was submitted to LOVD 3.0 shared installation (http://databases.lovd.nl/shared/) and received the variant ID # 0000064762.

### Variants prediction methods

In order to evaluate the deleterious consequence of putative variants, the following prediction software were used: Align GVGD [17], SIFT [18], Mutation Taster [19] and PolyPhen2 [20]. Grantham score [21] was obtained. The interspecies conservation of amino-acid was evaluated and the presence of variants was searched in 1000 genomes (http://www.1000genomes.org/) and Exome Variant Server (http://evs.gs.washington.edu/EVS/).

### Structural interpretation of the actin and myosin variants

The interpretation of the structural consequences of specific actin and myosin variants was performed by analyzing the recently published Rigor Actin-Tropomyosin-Myosin complex (PDB accession codes 4A7F, 4A7H, 4A7L and 4A7N), reported as the first subnanometer-resolution structure of the actin-tropomyosin-myosin complex in the rigor (nucleotide-free) state determined by cryo-EM [22]. Images were prepared using the Molmol software [23].

## Results

### Clinical analysis

**Fig 1:**
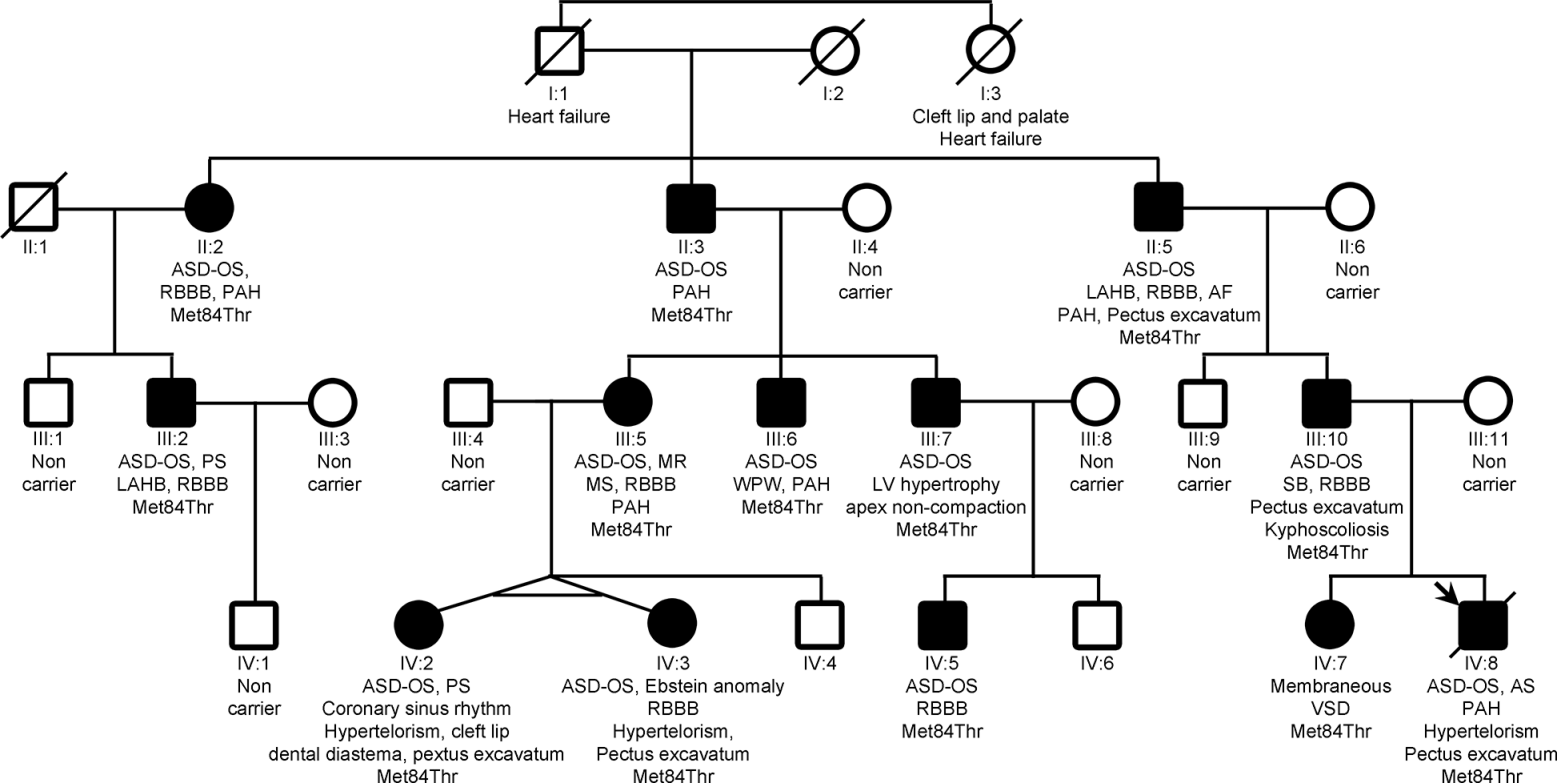
Pedigree of the family with recurrent cardiopathies. Squares (males) and circle (females), crossed symbols (deceased), empty symbols (unaffected) and filled symbols (affected), arrow (proband). AF: atrial fibrillation; AR: aortic regurgitation; AS: aortic stenosis; ASD-OS: atrial septal defect ostium secundum type; LAH: left anterior hemiblock; LV: left ventricle; PAH: pulmonary artery hypertension; PS: pulmonary stenosis; RBBB: right bundle branch block; SB: sinus bradycardia; VSD: ventricular septal defect; WPW: Wolff Parkinson White.

This Lebanese family belongs to the Christian Maronite community. It had 13 members (Fig 1) with congenital heart defect (CHD): 7 isolated atrial septal defects (ASD) (II:2, II:3, II:5, III:6, III:7, III:10 and IV:5), 2 ASD and pulmonary stenosis (PS) (III:2 and IV:2), 1 ASD and aortic stenosis (AS) (IV:8), 1 ASD and mitral regurgitation (MR) and stenosis (MR) (III:5), 1 ASD and Ebstein anomaly (IV:3) and 1 membranous ventricular septal defect (VSD) (IV:7). In addition, one family member had a Wolff-Parkinson-White syndrome (WPW) and had complained since childhood about short bouts of undocumented paroxysmal tachycardia (III:6), one affected relative had sinus bradycardia (III:10), and one had atrial fibrillation at the age of 40 years (II:5). Beside cardiac anomalies, 6 relatives had a midline defect: 5 cases of pectus excavatum (II:5, III:10, IV:2, IV:3 and IV:8), 1 case of kyphoscoliosis (III:10), 3 cases had hypertelorism (IV:2, IV:3 and IV:8), and 1 case had cleft lip and diastema between superior incisors (IV:2). All other family members had neither cardiac nor midline anomaly. The founder of the family (I:1) died at the age of 66 years of chronic heart failure although he had neither coronary disease nor left ventricle obstruction. He had a sister (I:3) who had a cleft lip and palate and died of heart failure at age of 60. This pedigree was reported earlier [24] (OMIM #603642: atrial septal defect, secundum, with various cardiac and noncardiac defects).

### Linkage analysis and gene mutation identification

The screening of the *NKX2-5* and *GATA4* genes in the proband DNA (IV:8) discovered no mutations. A set of 399 poly(AC) markers was used to genotype all available DNA of this family (13 affected and 9 unaffected individuals) (Fig 1). After a parametric analysis, we found that there were only 3 peaks above 0 (S1A Fig). The tallest peak reached a lod score of 2.98. It is located on chromosome 15. This result was robust to re-analysis with decreased penetrance values (to 85%). With non parametric parameters, there was a peak nearly exclusive on chromosome 15 (S1B Fig). Haplotypes of the chromosome 15 region obtained from linkage analysis demonstrated that all affected relatives had the same allele on the marker D15S1007 which is located at genomic position 33,545,560–33,545,736 (GRCh38) on the long arm of chromosome 15 at the q14 band (S2 Fig). Moreover, a recombination between D15S165 and D15S1007 on the centromeric side (individual II:3) and one between D15S1007 and D15S1012 on the telomeric side (individual IV:8) gave the limits of the genomic interval for the causal mutation (D15S165 to D15S1012). This genomic interval of about 7,747,000 nucleotides includes 37 coding genes among which the *ACTC1* gene is found. This gene encodes alpha cardiac actin 1. Since this gene appeared as the best candidate, the 6 coding exons were sequenced. A single heterozygous variant was found in the 3^rd^ exon: c.251T>C changing the Methionine at position 84 to a Threonine. This variant is absent in 1000 genomes and in the Exome Variant Server. The Met84 is highly conserved across species to yeast and the physico-chemical properties of Methionine and Threonine are significantly different (Grantham score of 81 on a range of 0 to 215). Three software (GVGD, SIFT, Mutation Taster) predicted that it is a disease causing variant but PolyPhen2 predicted the variant to be benign. This variant was found in all affected individuals and was absent from all unaffected relatives (Fig 1). Taken together these data, we concluded that this variant is the causal mutation.

### 3D structure analysis

**Fig 2:**
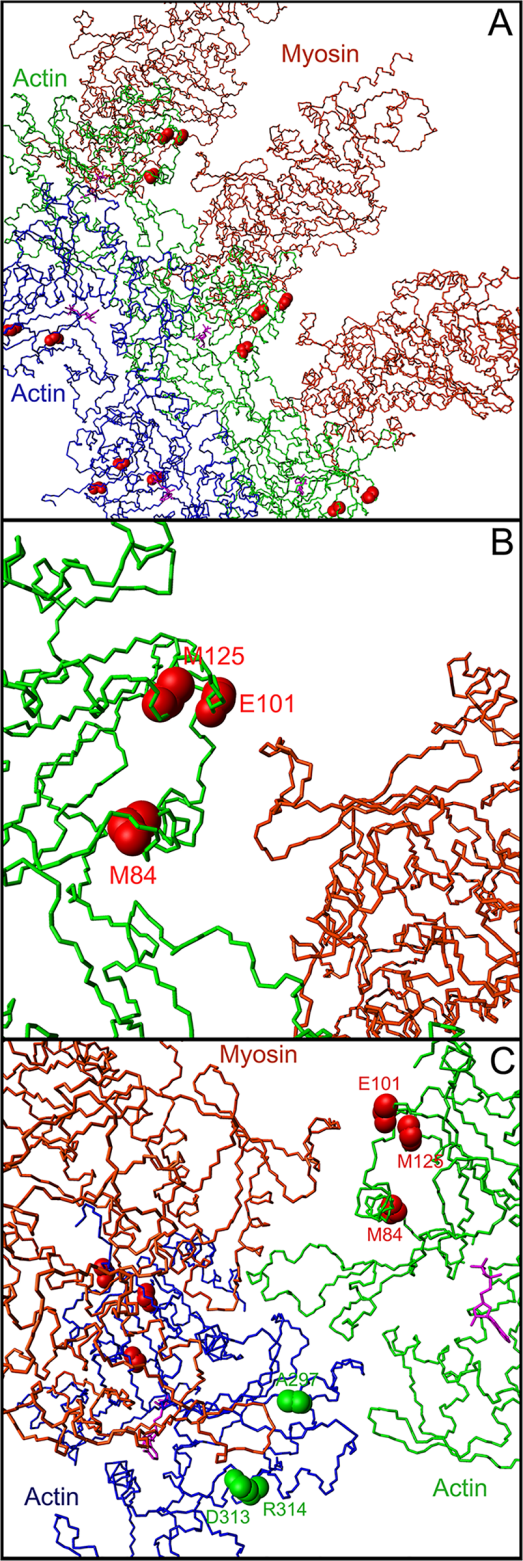
Molecular representation of the PDB 4A7L complex, displaying an actin fiber (blue and green monomers, with ADP molecules in purple) with myosin heads (brown monomers). Actin and myosin amino acid numbers are according to human numbering **(A)** Backbone representation of an actin fiber in complex with 3 myosin heads, showing secondary structure elements. The backbone atoms of the 3 amino acids altered by mutations causing atrial septal defects (residues 84, 101, and 125) are shown by red spheres on every actin monomer. **(B)** Close up on the interaction between the region spanning residues 84, 101 and 125 of an F-actin monomer and a very close loop of the myosin head. The actin monomer is shown in green, the myosin head is shown in brown. The interatomic distances measured in the complex between residues 84, 101 and 125 and the myosin surface typically range from 3 to 10 Å. The 562–571 region of the myosin head makes numerous contacts with the surface of the actin filament, and interacts closely with residues 84, 101 and 125 on the surface of actin. **(C)** Close up showing one myosin head interacting with the region 84, 101, and 125 region of the actin monomer, and the 297, 313 and 314 region of an adjacent actin monomer. The 562–571 region of the myosin head closely interacts with residues whose mutation leads to atrial septal defects (84, 101, and 125, in red), whereas the 367–365 region (human numbering) of the same myosin head interacts directly with an adjacent actin monomer (residues 297, 313, 314, in green), whose mutation leads to cardiomyopathies. The orientation of the actin monomers in panel A and B is similar whereas the molecules in panel C have been rotated for a better view of the interaction with residues 297, 313, and 314.

The Met84 residue is found within a surface exposed helix of the globular part of F-Actin. Interestingly, as observed in all the previously published F-actin fiber structures, there is no evidence of a native actin-actin contact surface involving residue 84 in actin filaments reconstructions (Fig 2A). Looking closely at the 3D structure of globular actin, this mutation appeared to reside in a region of the actin filament in extremely tight apposition to the myosin head (Fig 2B). Furthermore, two other well-characterized mutations of actin (p.(Glu101Lys) and p.(Met125Val)), which both also resulted mainly in atrial septal defects [9,25,26] are located in the same subdomain (Fig 2B). Disease-causing mutations disrupt both electrostatic and hydrophobic contacts, thereby directly perturbing the interaction between actin, tropomyosin and myosin. All published *ACTC1* mutations in humans are summarized on Table 1. Note that in several publications, the amino acid position was given after subtraction of the first two residues that are removed during actin maturation (the original reported Met123Val mutation should actually be described as Met125Val). Interestingly, *ACTC1* mutations resulting in congenital heart defects (essentially atrial septal defects) are restricted to the first half of the protein (from residue Met84 to residue Met178 [27]). Beyond residue Met178, all reported ACTC1 mutations result in diverse cardiomyopathies [10,28–31] with an unusual prevalence of non-compaction of the apex or hypertrophied apex.

**Table 1:** Reported cases with *ACTC1* gene mutations.

Familial/de novo	Cardiac Disease	Cardiac rythm	Evolution	Other features	Mutation	ENST00000290378 NM_005159.4	ENST00000290378 NM_005159.4	Reference
Familial (13x)	12x ASD, 1x VSD, 2x PS, 1x AR, 1x AS, 1x Ebstein	2x WPW, 1x AF, 1x SB	CHF	5x midline defects	p.(Met84Thr)	p.(Met84Thr)	c.251T>C	Current study
Familial (2x)	1x ASD, 1x VSD?	-	-	-	17bp del starting AA 86	p.(Pro72Hisfs*18)	c.215_231del	Matsson et al. 2008
Familial (18x)	5x hypertrophied and 3x trabeculated LV apex, 11x HCM, 4x MR, 2x AR	3x AF, 3x AV block or SB, 2x short PR interval	AF, CHF	-	p.(Glu99Lys)	p.(Glu101Lys)	c.301G>A	Arad M et al. 2005
Familial (94x)	22x apical HCM, 23x LVNC, 8x ASD, 1x VSD, 3x RCM	3x AF	1x CHF, 1x graft, 5x SD	-	p.(Glu99Lys)	p.(Glu101Lys)	c.301G>A	Monserrat L et al. 2007
Familial (9x)	6x HCM, 2x trabeculated and 5x hypertrophied LV apex, 1x ASD	1x VT, 2x VF	2x SD	-	p.(Glu99Lys)	p.(Glu101Lys)	c.301G>A	Olson T al. 2000
Familial (20x)	20x ASD	-	-	-	p.(Met123Val)	p.(Met125Val)	c.373A>G	Matsson H et al. 2008
de novo (17 mo)	HCM, hypertrophied LV apex	PM	-	-	p.(Pro164Ala)	p.(Pro166Ala)	c.496C>G	Olson et al. 2000.
Familial (6x)	6x ASD	-	-	-	p.(Met178Leu)	p.(Met178Leu)	c.532A>T	Greenway at al. 2014
Familial (3x)	2x HCM	-	1x SD	-	p.(Ala232Val)	p.(Ala232Val)	c.695C>T	Van Driest et al. 2003
Familial (18x)	13x HCM, 5x MR, 2x AR	1x AF, 1x WPW, 1x VF	2x DCM	-	p.(Ala295Ser)	p.(Ala297Ser)	c.889G>T	Mogensen et al. 1999
Familial (3x)	2x RCM, 1x RMC/DCM	-	1x graftn	-	p.(Asp313His)	p.(Asp313His)	c.937G>C	Kaski et al. 2008
Familial (4x)	3x DCM	-	-	-	p.(Arg312His)	p.(Arg314His)	c.941G>A	Olson et al. 1998
de novo (8 yo)	HCM, hypertrophied LV apex	VF, IAD	-	-	p.(Ala331Pro)	p.(Ala333Pro)	c.997G>C	Olson et al. 2000
Familial (4x)	4x DCM	-	-	-	p.(Glu361Gly)	p.(Glu363Gly)	c.1088A>G	Olson et al. 1998

In the first column (familial/sporadic), the number of reported cases is notified (for instance: 13x: 13 reported cases). In de novo mutations, the age at diagnosis is stated. Mutation: mutation description as reported in the original article; Columns “ENST00000290378, NM_005159.4”: current description at the protein and DNA levels of the same mutation as in the column “Mutation”. AF: Atrial Fibrillation, AR: Aortic Regurgitation, AS: Aortic Stenosis, ASD: Atrial Septal Defect, AV: Atrioventricular, CHF: Cardiac Heart Failure, DCM: Dilated Cardiomyopathy, HCM: Hypertrophic Cardiomyopathy, IAD: Implantable Automatic Defibrillator, LV: Left Ventricle, LVNC: Left Ventricular Non Compaction, mo: months old, MR: Mitral Regurgitation, PM: Pace Maker, PS: Pulmonary Stenosis, RCM: Restrictive Cardiomyopathy, SB: Sinus Bradycardia, SD: Sudden Death, VF: Ventricular Fibrillation, VSD: Ventricular Septal Defect, VT: Ventricular Tachycardia, WPW: Wolff Parkinson White, yo: years old.

## Discussion

The list of genes involved in congenital heart defects (CHD) is growing [1]. At the same time, the frontier between CHD and cardiomyopathies (CM) is becoming blurred. There are reports on familial cases with relatives being affected with CM or CHD or both. In addition, the list of genes which can lead to CM and/or CHD is also growing [2–8,11,12]. The actin gene is one of these genes. *ACTC1* mutations were first identified in a series of dilated CM [30]. Other mutations can result in apical hypertrophic CM, while others are associated with CHD and in particular atrial septal defects (ASD). In this report, mutation carriers of the p.(Met84Thr) missense suffer from a variety of CHD (ASD, Ebstein anomaly, VSD) but also from valvular anomalies (aortic and pulmonary stenosis, mitral regurgitation and stenosis), conduction tissue anomalies (sinus bradycardia, WPW syndrome), and CM (hypertrophic CM in one patient and presumably dilated CM in the founder and his sister). Thus, alpha cardiac actin 1 mutations can result in a variety of cardiopathies even within a single family suggesting that modifying factors might modulate the expressivity of *ACTC1* mutation.

In addition to cardiac signs, several patients showed midline anomalies (diastema of the upper incisors, cleft lip, hypertelorism, kyphoscoliosis and pectus excavatum). Midline defects are very rare and the occurrence of 5 cases (6 cases if the grand-aunt I:3 is included) in a single family cannot be fortuitous. In addition, each relative with a midline defect actually has more than one midline defect sign. The co-segregation of midline defects with cardiac anomalies and the Met84Thr mutation suggests that cardiac and midline defect could be secondary to the *ACTC1* Met84 mutation. The co-occurrence of cardiac and midline defects was never reported previously in *ACTC1* mutation carriers. Alternatively, it is possible that a second mutation in another gene is present in this minimal genomic region. Actually, there exists a single gene among the 36 genes—excluding the *ACTC1* gene–(S1 Table) which can lead to midline defects. This gene, *SLC12A6*, leads to mild midline defects similar to the ones reported in this study. However, it also leads to mental disability, complete or partial agenesis of the corpus callosum, and severe peripheral neuropathy [32]. This severe disease is autosomal recessive, so we can rule out this type of inheritance in this Lebanese family because individuals with midline defect share only one parental haplotype as evidenced by linkage analysis. This report prompts cardiologists to pay attention to midline anomalies in familial ASD. It is possible that cardiologists have overlooked midline defects and failed to report this type of anomaly in previous *ACTC1* mutation reports.

The actin p.(Met84Thr) mutation, which was identified in this work, as well as the previously identified p.(Glu101Lys) [9,25] and p.(Met125Val) [26] mutations, result mainly in ASD. Those 3 mutations occur in a small spatially well-defined region, which is on a surface exposed region of F-actin in very close contact with myosin monomers, as observed in the most accurate muscular fiber reconstruction published to date. The importance of this region for the Actin-Myosin interaction is supported by previous mutagenesis studies demonstrating that altering the charge of residue Glu101 by histidine substitution reduces in vitro motility by five-fold [33] whereas the p.(Met125Val) substitution showed a significantly reduced affinity for myosin [26]. Another argument to support the importance of the tight actin-myosin interaction is found by carefully analyzing the position of residues 297 [29], 313 [28] and 314 [30] of actin in the 4A7L actin-myosin complex. Mutations on those residues have been known to lead to various cardiomyopathies and they also appear to make extremely close contact with the adjacent myosin monomer, but using a totally different interaction surface compared to residues 84 (current study), 101 [9,10,25] and 125 [26] (Fig 2C).

The human *ACTC1* gene produces a protein with 94% homology to the gamma actin gene (*ACTG1*). Mutations in this latter gene were associated with dominant progressive deafness [34], a disease that displays sensorineural hearing loss beginning in the twenties in the high frequencies and steadily progressing to include all frequencies. Although the two non-muscular actin genes (*ACTG1* and *ACTB*) are expressed concomitantly in all mammalian cells, the auditory hair cell is one of the rare cell types where the predominant isoform is gamma actin (*ACTG1*). In auditory hair cells, the gamma actin protein is found in stereocilia, the cuticular plate, and adherens junctions. One of the six mutations found in the initial report is a threonine to isoleucine change at position 89, a position very close to the congenital cardiac disease *ACTC1* mutations. Although the gamma actin residue at the corresponding site is not a threonine but a valine, it is interesting to comment on the changes induced by this p.(Thr89Ile) missense variant. It was tested in the yeast *Saccharomyces cerevisiae* because yeast actin is 91% identical to human gamma actin and the Thr89 residue is conserved in both species. The Thr89Ile mutation resulted in a higher population of cells with fragmented and/or depolarized cables and uniform distribution of patches in both mother cell and bud in comparison to wild-type [35]. However, the *in vitro* ability of purified Thr89Ile mutant actin to polymerize was not grossly modified suggesting an altered *in vivo* interaction with one or more of the numerous acting-binding proteins known to control actin cytoskeletal function and dynamics. It was actually the case since the p.(Thr89Ile) variant F-actin is much more susceptible to cofilin disassembly than wild type actin [36]. Cofilin severs F-actin and sequesters actin monomers. This result suggests that the Thr89 residue is involved in non-actin protein interaction and that mutations in this region destabilize protein interaction with cofilin in a presumably similar way as the cardiac actin Met84 mutation might destabilize the actin/myosin interaction.

In conclusion, we reported a novel *ACTC1* gene mutation which resulted in various congenital heart defects and arrhythmia. This family study suggested that *ACTC1* mutation could also lead to midline defects. Finally, we provided evidence to possibly explain the pleiotropic consequences of *ACTC1* gene mutations by pointing to particular molecular domains where actin and myosin heavy chain are in close contact. Depending on the domain, *ACTC1* mutation can lead rather to congenital heart defects or to cardiomyopathies.

## Supporting Information

S1 Fig Parametric (A) and non parametric (B) lod score analyses. (DOCX)

S2 Fig Pedigree of the family with cardiopathies. (DOCX)

S1 Table Summary of the 37 encoding genes included in the genomic interval inherited in all affected individuals. (DOCX)
