# Supplementary material for: A Novel Alpha Cardiac Actin (ACTC1) Mutation Mapping to a Domain in Close Contact with Myosin Heavy Chain Leads to a Variety of Congenital Heart Defects, Arrhythmia and Possibly Midline Defects
Source: PLoS One. 2015 Jun 10;10(6):e0127903. doi: 10.1371/journal.pone.0127903 (PMC4464657; doi:10.1371/journal.pone.0127903)
Supplement: S1 Fig — (DOCX) [file pone.0127903.s001.docx]

**SUPPORTING INFORMATION: Manuscript PONE-D-15-0835R1**

**S1 Fig. A and B**: Parametric and non parametric lod score analyses


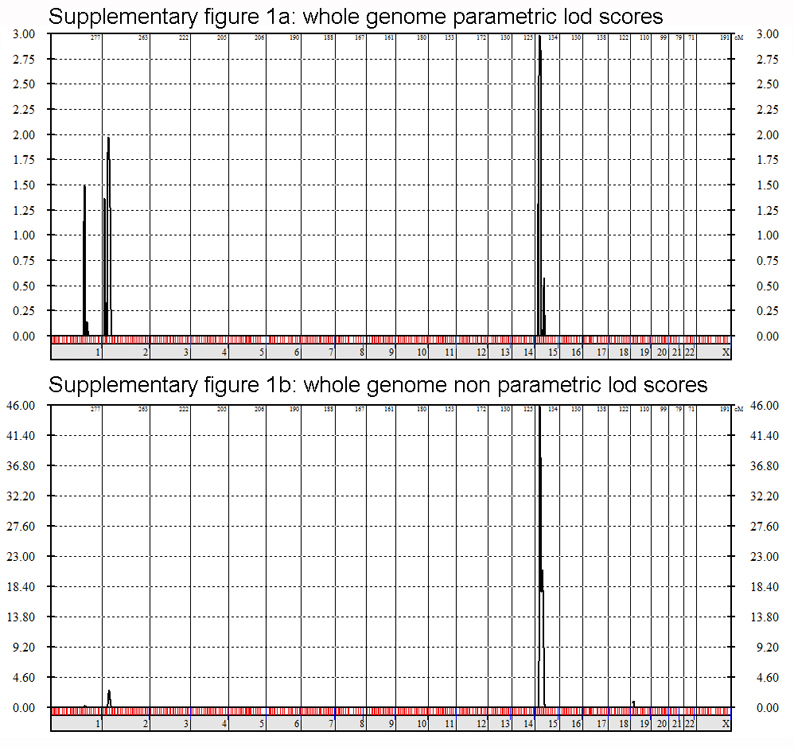


**S1 Fig. A and B legend**

(**a**) whole genome calculation of parametric lodscores (mutation prevalence: 0.01%), penetrance: 100%, phenocopy rate: 0%). The lod score values are indicated on the Y axis, the chromosomal positions on the X axis from chromosome 1 to chromosome X. The maximum lod score is obtained on chromosome 15 (2.98) while there exists only a minor peak on chromosome 1 and 2. The peak lod score remained at 2.98 up to a penetrance value of 85%. (**b**) whole genome calculation of non parametric lod scores (there is no hypothesis made on mutation prevalence, penetrance, phenocopy rate and inheritance type). There is a single peak on chromosome 15. The non-parametric lod score values are indicated on the Y axis the chromosomal positions on the X axis from chromosome 1 to chromosome X.
