# Supplementary material for: A Novel Alpha Cardiac Actin (ACTC1) Mutation Mapping to a Domain in Close Contact with Myosin Heavy Chain Leads to a Variety of Congenital Heart Defects, Arrhythmia and Possibly Midline Defects
Source: PLoS One. 2015 Jun 10;10(6):e0127903. doi: 10.1371/journal.pone.0127903 (PMC4464657; doi:10.1371/journal.pone.0127903)
Supplement: S2 Fig — (DOCX) [file pone.0127903.s002.docx]

**S2 Fig.**: Pedigree of the family with cardiopathies.


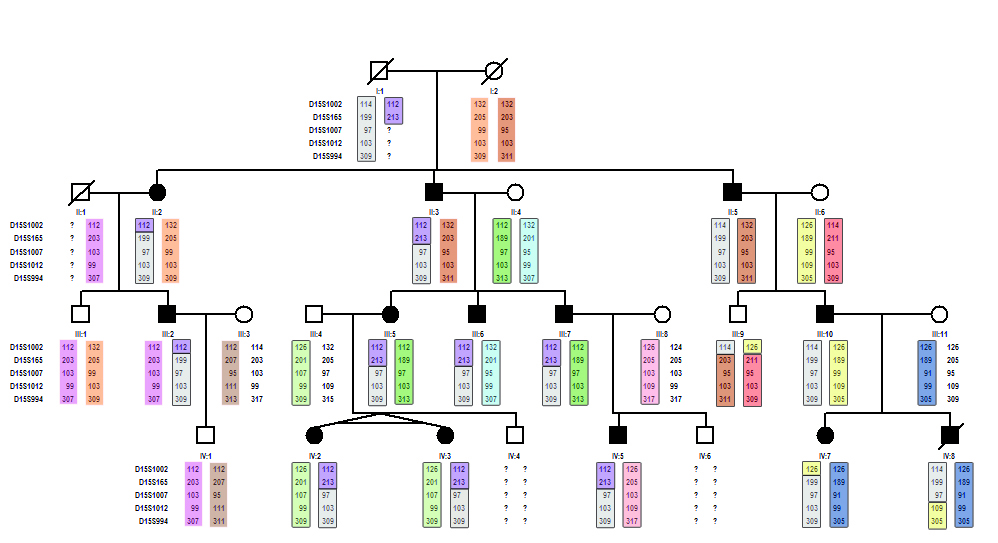


**S2 Fig. Legend**

The segregations of the 5 poly(AC) markers of the chromosomal region with a peak on chromosome 15 are indicated. Haplotypes are highlighted with colors. Note that all affected individuals received the allele 97 of marker D15S1007. The haplotype carrying the ACTC1 mutation is presented in light grey. Allele numbers are actually amplicons sizes in nucleotides. The haplotypes of individuals I:1, I:2 and II:1 were inferred.
