# Supplementary material for: A Novel Alpha Cardiac Actin (ACTC1) Mutation Mapping to a Domain in Close Contact with Myosin Heavy Chain Leads to a Variety of Congenital Heart Defects, Arrhythmia and Possibly Midline Defects
Source: PLoS One. 2015 Jun 10;10(6):e0127903. doi: 10.1371/journal.pone.0127903 (PMC4464657; doi:10.1371/journal.pone.0127903)
Supplement: S1 Table — (DOCX) [file pone.0127903.s003.docx]

**S1Table**: Summary of the 37 encoding genes included in the genomic interval inherited in all affected individuals.

| **Ensembl Gene ID** | **Gene name** | **Phenotype description** | **Ensembl Family Description** | **Description** |
| --- | --- | --- | --- | --- |
| ENSG00000166912 | MTMR10 |  | MYOTUBULARIN RELATED | myotubularin related protein 10 |
| ENSG00000134160 | TRPM1 | Congenital stationary night blindness | TRANSIENT RECEPTOR POTENTIAL CATION CHANNEL SUBFAMILY M MEMBER LONG TRANSIENT RECEPTOR POTENTIAL CHANNEL | transient receptor potential cation channel, subfamily M, member 1 |
| ENSG00000169926 | KLF13 |  | KRUEPPEL FACTOR 9 BASIC TRANSCRIPTION ELEMENT BINDING 1 BTE BINDING 1 GC BOX BINDING 1 TRANSCRIPTION FACTOR BTEB1 | Kruppel-like factor 13 |
| ENSG00000169918 | OTUD7A |  | OTU DOMAIN CONTAINING 7B EC_3.4.19.12 CELLULAR ZINC FINGER ANTI NF KAPPA B ZINC FINGER A20 DOMAIN CONTAINING 1 ZINC FINGER CEZANNE | OTU deubiquitinase 7A |
| ENSG00000175344 | CHRNA7 | 15q13.3 microdeletion syndrome | ALPHA7 NICOTINIC RECEPTOR FRAGMENT | cholinergic receptor, nicotinic, alpha 7 (neuronal) |
| ENSG00000249931 | GOLGA8K |  | GOLGIN SUBFAMILY A MEMBER | golgin A8 family, member K |
| ENSG00000206127 | GOLGA8O |  | GOLGIN SUBFAMILY A MEMBER | golgin A8 family, member O |
| ENSG00000232653 | GOLGA8N |  | GOLGIN SUBFAMILY A MEMBER | golgin A8 family, member N |
| ENSG00000198826 | ARHGAP11A |  | RHO GTPASE ACTIVATING RHO TYPE GTPASE ACTIVATING | Rho GTPase activating protein 11A |
| ENSG00000166922 | SCG5 |  | NEUROENDOCRINE 7B2 PRECURSOR SECRETOGRANIN V SECRETOGRANIN 5 SECRETORY GRANULE ENDOCRINE I [CONTAINS N TERMINAL PEPTIDE; C TERMINAL PEPTIDE] | secretogranin V (7B2 protein) |
| ENSG00000166923 | GREM1 | Hereditary mixed polyposis syndrome | GREMLIN 1 PRECURSOR CYSTEINE KNOT SUPERFAMILY 1 BMP ANTAGONIST 1 | gremlin 1, DAN family BMP antagonist |
| ENSG00000248905 | FMN1 |  | FORMIN | formin 1 |
| ENSG00000198838 | RYR3 |  | RYANODINE RECEPTOR RYR MUSCLE CALCIUM RELEASE CHANNEL MUSCLE TYPE RYANODINE RECEPTOR TYPE RYANODINE RECEPTOR | ryanodine receptor 3 |
| ENSG00000169857 | AVEN |  | CELL DEATH REGULATOR AVEN | apoptosis, caspase activation inhibitor |
| ENSG00000184984 | CHRM5 |  | MUSCARINIC ACETYLCHOLINE RECEPTOR | cholinergic receptor, muscarinic 5 |
| ENSG00000134153 | EMC7 |  | ER MEMBRANE COMPLEX SUBUNIT 7 PRECURSOR | ER membrane protein complex subunit 7 |
| ENSG00000182405 | PGBD4 |  | PIGGYBAC TRANSPOSABLE ELEMENT DERIVED 4 | piggyBac transposable element derived 4 |
| ENSG00000134152 | KATNBL1 |  | KATNB1 1 KATANIN P80 SUBUNIT B 1 | katanin p80 subunit B-like 1 |
| ENSG00000128463 | EMC4 |  | ER MEMBRANE COMPLEX SUBUNIT 4 TRANSMEMBRANE 85 | ER membrane protein complex subunit 4 |
| ENSG00000140199 | SLC12A6 | Agenesis of the corpus callosum with peripheral neuropathy (ACCPN) | SOLUTE CARRIER FAMILY 12 MEMBER ELECTRONEUTRAL POTASSIUM CHLORIDE COTRANSPORTER K CL COTRANSPORTER | solute carrier family 12 (potassium/chloride transporter), member 6 |
| ENSG00000182117 | NOP10 | Dyskeratosis congenita | UNKNOWN | NOP10 ribonucleoprotein |
| ENSG00000184507 | NUTM1 |  | NUT FAMILY MEMBER | NUT midline carcinoma, family member 1 |
| ENSG00000176454 | LPCAT4 |  | LYSOPHOSPHATIDYLCHOLINE ACYLTRANSFERASE LPC ACYLTRANSFERASE LPCAT LYSOPC ACYLTRANSFERASE EC_2.3.1.23 1 ACYLGLYCEROPHOSPHOCHOLINE O ACYLTRANSFERASE 1 ALKYLGLYCEROPHOSPHOCHOLINE O ACETYLTRANSFERASE 67 ACETYL COA:LYSO PLATELET ACTIVATING FACTOR ACETYLTRANSFE | lysophosphatidylcholine acyltransferase 4 |
| ENSG00000175265 | GOLGA8A |  | GOLGIN SUBFAMILY A MEMBER | golgin A8 family, member A |
| ENSG00000215252 | GOLGA8B |  | GOLGIN SUBFAMILY A MEMBER | golgin A8 family, member B |
| ENSG00000159248 | GJD2 |  | GAP JUNCTION DELTA 2 CONNEXIN GAP JUNCTION ALPHA 9 | gap junction protein, delta 2, 36kDa |
| ENSG00000159251 | ACTC1 | Cardiomyopathy dilated type 1R (CMD1R), hypertrophic type II (CMH11), atrial septal defect type 5 (ASD5) | ACTIN | actin, alpha, cardiac muscle 1 |
| ENSG00000021776 | AQR |  | INTRON BINDING AQUARIUS | aquarius intron-binding spliceosomal factor |
| ENSG00000198146 | ZNF770 |  | ZINC FINGER 770 | zinc finger protein 770 |
| ENSG00000134146 | DPH6 |  | DIPHTHINE AMMONIA LIGASE EC_6.3.1.14 ATP BINDING DOMAIN CONTAINING 4 DIPHTHAMIDE SYNTHASE DIPHTHAMIDE SYNTHETASE DPH6 HOMOLOG | diphthamine biosynthesis 6 |
| ENSG00000186073 | C15orf41 | Congenital dyserythropoietic anemia type I | UNCHARACTERIZED | chromosome 15 open reading frame 41 |
| ENSG00000134138 | MEIS2 |  | HOMEOBOX | Meis homeobox 2 |
| ENSG00000166069 | TMCO5A |  | TRANSMEMBRANE AND COILED COIL DOMAIN CONTAINING | transmembrane and coiled-coil domains 5A |
| ENSG00000166068 | SPRED1 | Legius syndrome | SPROUTY RELATED EVH1 DOMAIN CONTAINING SPRED | sprouty-related, EVH1 domain containing 1 |
| ENSG00000171262 | FAM98B |  | FAM98A | family with sequence similarity 98, member B |
| ENSG00000172575 | RASGRP1 | Systemic lupus erythematosus | RAS GUANYL RELEASING CALCIUM AND DAG REGULATED GUANINE NUCLEOTIDE EXCHANGE FACTOR CALDAG | RAS guanyl releasing protein 1 (calcium and DAG-regulated) |
| ENSG00000175779 | C15orf53 |  | UNCHARACTERIZED | chromosome 15 open reading frame 53 |
